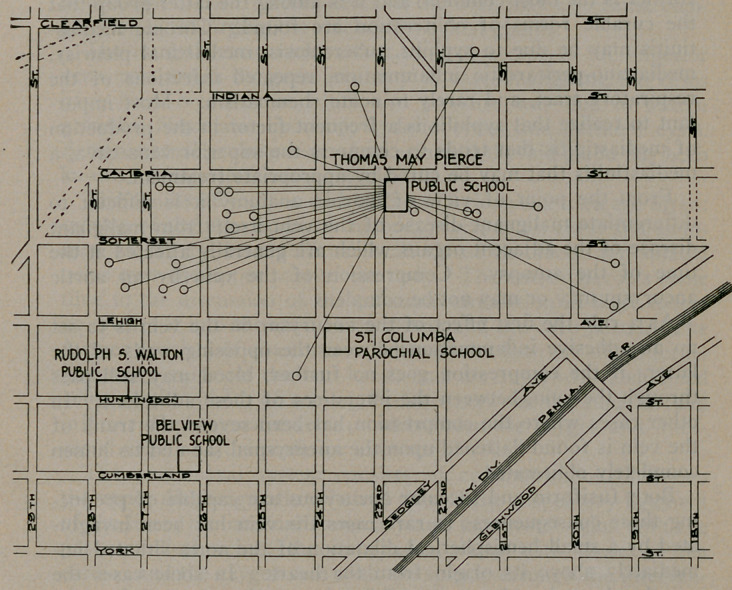# Role of the School in Spread of Scarlet Fever

**Published:** 1914-02

**Authors:** 


Role of the School in Spread of Scarlet Fever. Walter W. Roach, *Am. Jour. Pub. Health,* Vol. 2, No. 6, describes, with map loaned by courtesy of the Editor, an epidemic occurring in Philadelphia, January-March, 1913. On inspection of the schools in March, seven desquamating cases were found.

## Figures and Tables

**Figure f1:**